# Magma chamber evolution during the 1650 AD Kolumbo eruption provides clues about past and future volcanic activity

**DOI:** 10.1038/s41598-020-71991-y

**Published:** 2020-09-22

**Authors:** K. I. Konstantinou

**Affiliations:** Department of Earth Sciences, National Central University, Jhongli, 320 Taiwan

**Keywords:** Volcanology, Natural hazards

## Abstract

Kolumbo submarine volcano lies 7 km NE of Santorini caldera and its last eruption which occurred in 1650 AD, caused damage and casualties to the nearby islands. Here a simple model of a chamber, containing silicic magma underlain by a smaller quantity of mafic magma, is utilised in order to understand the chamber behaviour during the 1650 AD eruption. Results show that in order to reproduce the duration (83–281 days) and the dense rock equivalent volume (${\sim }\,2\, \hbox {km}^3$) of the eruption, initial overpressure in the chamber should be around 10 MPa and the mafic magma should occupy up to 5% of the chamber volume. It is found that the time needed to inject mafic magma equal to 1–15% of the chamber volume varies between 1.4–13.7 ka, if the radius of the chamber is about 1500 m as inferred from tomographic images. These long recurrence times agree well with the small number of eruptions ($N = 5$) within a period of > 70 ka and suggest that an eruption in the near future is unlikely. Volcanic activity at Kolumbo is probably triggered by a combination of exsolved volatiles and a small but steady influx of mafic melt in the chamber.

## Introduction

The formation, growth and evolution of magma chambers represent fundamental issues in volcanological studies and constitute the basis for the formulation of models of how volcanoes erupt^1–4^. Magma chambers residing in the upper crust (under lithostatic pressure of 200 ${\pm }$ 50 MPa) usually contain evolved magma which consists of variable amounts of melt, crystals and volatiles collectively known as ‘mush’^5^. A common mechanism for triggering an eruption in such a chamber is the injection of high temperature, low viscosity mafic magma at its base^6–8^. The injection has the effect of not only generating overpressure by adding more material in the magma chamber, but also heating the evolved magma and remobilising it. Evidence for this triggering mechanism comes from petrological observations in several arc volcanoes around the world that reveal different degrees of mixing of the two magma types in erupted products^9^.

**Figure 1:**
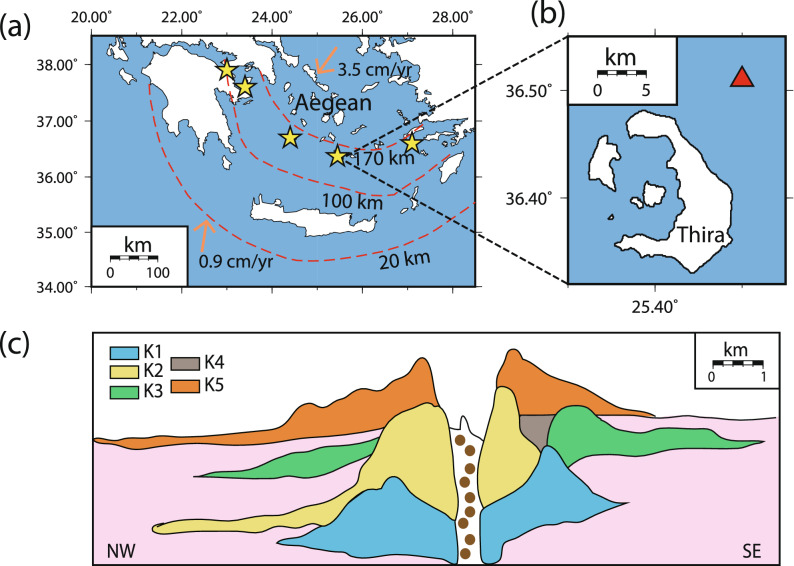
(**a**) Map of the southern Aegean showing the distribution of arc volcanoes (yellow stars), the isodepths of the Wadati–Benioff zone (after Ref.^11^), as well as the motion vectors of the African lithosphere and the Aegean upper plate (after Ref.^10^). (**b**) A magnified map of the area around the Santorini caldera where the location of the Kolumbo submarine volcano is shown as a red triangle. (**c**) Cross-section across the Kolumbo volcano edifice that depicts the succession of five layers (K1–K5) that have been deposited during volcanic activity (after Ref.^15^). The different parts of the Figure have been prepared using Generic Mapping Tools (GMT, http://gmt.soest.hawaii.edu), CorelDraw (https://www.coreldraw.com) and Adobe illustrator version CS6 (https://www.adobe.com/products/illustrator.html).

**Figure 2:**
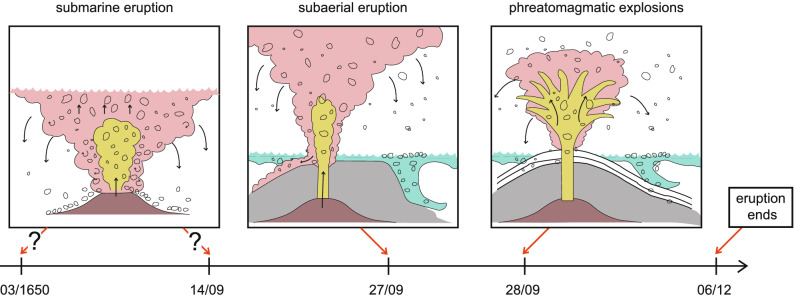
Cartoon that summarizes the different stages of the 1650 AD eruption of Kolumbo based on the study of Cantner et al.^17^. The red arrows point to the specific dates that mark the onset of each stage (see text for more details). The starting point of the submarine eruption could be March 1650 AD, or 14 September of the same year. The Figure was drafted using CorelDraw (https://www.coreldraw.com) and Adobe illustrator version CS6 (https://www.adobe.com/products/illustrator.html).

The southern Aegean hosts a chain of volcanoes that have been formed due to the subduction of the African lithosphere underneath the Eurasian plate^10,11^ (Fig. 1a). Kolumbo is one of them, located at a distance of 7 km from the island complex of Santorini caldera (Fig. 1b) with its crater area lying at a depth of 500 m below sea level. Even though Kolumbo is very close to Santorini, geochemical studies indicate that there is no petrogenetic link between the two magmatic systems^12^. Local earthquake tomography revealed the existence of a low velocity body at a depth of 5–7 km underneath Kolumbo^13^, a depth range which is also consistent with the distribution of earthquakes and the local stress field^14^. High-resolution seismic reflection profiles along its submarine edifice have imaged five distinct layers (K1–K5 in Fig. 1c) of eruption deposits^15^. Except from the top layer K5 which corresponds to the deposits of the 1650 AD eruption, the age of the other four layers is very uncertain and these may have accumulated over a period of > 70 ka or even up to 600 ka. The 1650 AD eruption was accompanied by pyroclastic flows and a tsunami that inundated the eastern coast of the island of Thira^16–18^. The presence of mafic enclaves in the evolved erupted products of the 1650 AD eruption as well as in the deposits of layer K2 suggests that the injection of mafic magma may have acted as a triggering mechanism in both eruptions^12,17^.

The eruption of Kolumbo in 1650 AD has been studied in detail by Cantner et al.^17^ who analysed its deposits in terms of petrography/geochemistry and suggested an interpretation for the physical processes that took place during the eruption. Here only a brief description of the course of events during the eruption as well as the key geochemical/petrological results will be given, referring the reader to Cantner et al.^17^ for more details. Already since March 1650 the inhabitants of the nearby islands started experiencing damaging earthquakes and subterranean roaring that reached a climax around 14 September of the same year. This time period probably corresponds to the pressurisation of the magma chamber and the formation of conduits that reached the seafloor, initiating the submarine stage of the eruption (Fig. 2). By 27 September the first plumes could be seen rising above the sea surface along with pumice rafts floating on the sea. The eruption at this point became subaerial, producing pumice that either separated from the plume and floated on the sea surface, or sank underneath generating vertical gravity flows. Explosions started occurring from 28 September onwards, while the eruption plume covered a large portion of the sky producing lightning and ejected incandescent rocks. This marks the onset of phreatomagmatic activity, where the hot magma came into contact with sea water at atmospheric conditions rather than near the seafloor where hydrostatic pressure is high (cf. Fig. 2). These phenomena continued to occur for more than 2 months, causing house roofs to collapse under the weight of the accumulated ash and the death of people/animals from inhalation of toxic fumes.

The eruption finally ended on 6 December 1650 when all phenomena subsided and the sea water became clear again. The duration of the eruption, as judged by the historical accounts described above, may vary from a minimum of 83 days (${\sim }$ 0.22 years) to a maximum of 281 days (${\sim }$ 0.76 years) depending on the choice of the starting date of the submarine eruption (March versus mid-September 1650). The bulk volume of the eruption deposits, inferred from the seismic reflection profiles around Kolumbo^15^, ranges from a minimum of 4.2 $\hbox {km}^3$ to a maximum of 6.3 $\hbox {km}^3$. Estimates of dense rock equivalent (DRE) volume yielded a value of 2 $\hbox {km}^3$ or slightly larger^19^.

Geological samples in the form of push/box cores and rocks from the crater wall of Kolumbo were obtained during several cruises in the area (for more information see Cantner et al.^17^). Geochemical analysis of the pumice samples revealed a rhyolitic composition with $\hbox {SiO}_2$ between 73.7–74.2 wt.%, indicating that the original magma was more evolved than the one that produced the pyroclastics of the nearby Santorini caldera. The volatile content was determined from melt inclusions and it was found to vary from 1.9 to 8.3 wt.% with a median value of 6.5 wt.% that serves as a conservative estimate. Pre-eruption temperature and equilibration pressure of the magma were found to be 750 $^{\circ }$C and 150 MPa respectively (for methodological details see Cantner et al.^17^). This pre-eruption pressure corresponds to a depth of about 5 km and correlates well with the location of the low-velocity anomaly found in tomographic images^13^. As mentioned earlier, a significant finding of the petrographic analysis was the presence of mafic inclusions in the eruption products that suggest the injection of mafic magma in the chamber prior to the eruption. Despite the fact that Kolumbo represents a source of significant volcanic hazard for the nearby islands, there has not been any study to-date that has modeled the magma chamber evolution during the 1650 AD eruption and has drawn conclusions about the long-term eruptive potential of this volcano. The present work tries to fill this gap by addressing three interrelated topics: (1) what were the conditions inside the magma chamber during the 1650 AD eruption, (2) how these conditions can affect the frequency, duration and size of eruptions, and (3) what might be the long-term eruptive behaviour of Kolumbo’s magma chamber. In the next sections the physical parameters summarised above will be used in order to model the evolution of the magma chamber and obtain answers to these questions.

**Figure 3:**
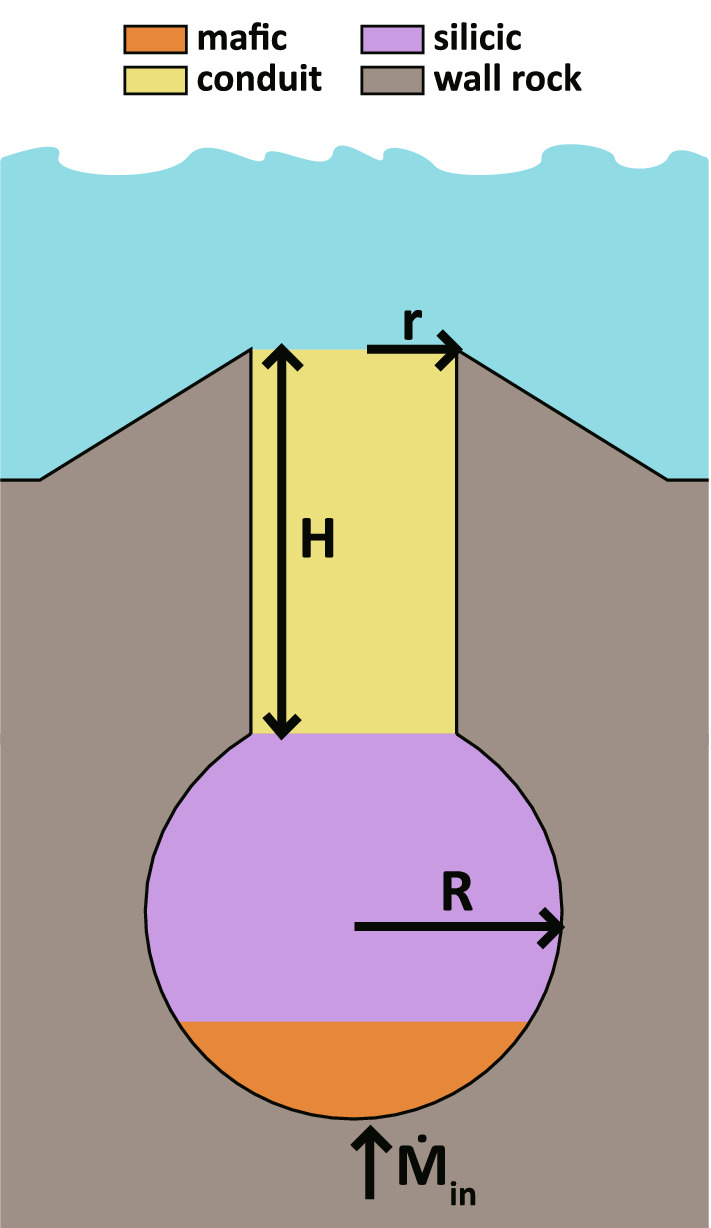
Cartoon that illustrates the geometrical configuration of the model magma chamber beneath Kolumbo (see text for more details).

## Results

### Simulations of the 1650 AD eruption

The model employed in this study assumes that the conditions in the chamber generate the necessary overpressure to drive a fracture to the surface and start the eruption. The magma chamber itself is considered as a sphere of volume *V* and radius *R* whose roof lies at a depth *H* below the seafloor while it is connected with a conduit of radius *r* (Fig. 3). The chamber contains a percentage ${\varphi }$ of mafic magma and the remainder is occupied by silicic magma of volume (1-${\varphi }$)*V*. Heat is then transferred from the hotter mafic to the cooler silicic magma, causing dissolution of its crystals and its remobilisation. The opposite process takes place in the mafic magma that is cooling and this causes crystallisation of minerals and saturation of the residual melt with volatiles. The model assumes thermodynamic and chemical equilibrium between the different phases and that bubbles and crystals stay in the layer in which they formed. Heat conducted to the wall rock around the chamber and the recharge rate of mafic magma are not included, since both processes affect the magma chamber over timescales much larger than the timescale of the eruption.

Some of the parameters required by the model can be constrained by the results summarised previously or by assuming reasonable, in a volcanological sense, values. The radius *R* of the magma chamber is taken equal to 1,500 m as inferred from local earthquake tomography^13^ and its depth *H* as 5 km following Cantner et al.^17^. The temperature of the silicic magma is assumed to be 750 $^{\circ }$C ($=$ 1,023 K) with a viscosity of 10$^7$ Pa s, while the mafic magma is considered to have a temperature of 1,200 $^{\circ }$C ($=$ 1,473 K). A lower temperature for the mafic magma of 1,050 $^{\circ }$C ($=$ 1,323 K) would have a negligible effect on the time variation of eruption rate and volume, but would slightly change the total volume of erupted material by 0.02–0.3 $\hbox {km}^3$. The silicic magma is assumed to be in a mushy state with a mass fraction of crystals equal to 0.4 which is slightly below the rheological lockup limit. The mafic magma is considered to be crystal free and to contain a mass fraction of volatiles equal to 4 wt.%. A list of parameters whose values have been adopted in this way can be found in Table 1. The remaining parameters, namely conduit radius *r*, initial overpressure ${\Delta }p_0$, percentage of mafic magma ${\varphi }$ and volatile ($\hbox {H}_2$O vapor) mass fraction, are varied so as to understand how they affect the duration and volume output of the eruption.

**Table 1:** List of the values that have been adopted for the model calculations described in the text (all symbols are defined in section “Methods”).

Quantity	Value
$T_m$	1,473 K
$T_s$	1,023 K
${\sigma }_{mls}$	2,300 kg/m$^3$
${\sigma }_{mlm}$	2,600 kg/m$^3$
${\sigma }_{cm}$	2,800 kg/m$^3$
${\sigma }_{cs}$	2,800 kg/m$^3$
$x_s$	0.4
$x_m$	0.0
${\beta }_m$	$1.8\times 10^{10}$ Pa
${\beta }_s$	$10^{10}$ Pa
${\beta }_w$	$10^{11}$ Pa
${\Gamma }_m$	$1.8248 \times 10^{-5}$K$^{-1}$
${\Gamma }_s$	$1.8248\times 10^{-5}$K$^{-1}$
*L*	$3\times 10^5$ J/kg
$Cp_m$	1,450 J/kg/K
$Cp_s$	1,680 J/kg/K
${\mu }_s$	10$^7$ Pa s
*R*	1,500 m
*H*	5,000 m

**Figure 4:**
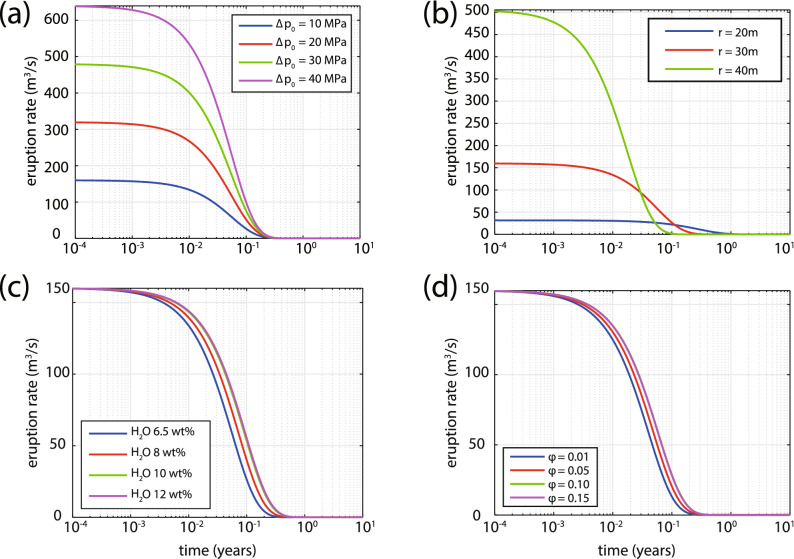
Summary of the model simulation results for the variation of eruption rate as a function of time for a range of: (**a**) initial overpressure (other parameters held constant: $r = 30$ m, ${\varphi } = 0.1$, 6.5 wt.% of $\hbox {H}_2$O), (**b**) conduit radius (other parameters held constant: ${\Delta }p = 10$ MPa, ${\varphi } = 0.1$, 6.5 wt.% of $\hbox {H}_2$O), (**c**) mass fraction of volatiles ($\hbox {H}_2$O vapor) of the silicic magma (other parameters held constant: $r = 30$ m, ${\varphi } = 0.1$, ${\Delta }p = 10$ MPa), (**d**) percentage of mafic magma that occupies the chamber (other parameters held constant: $r = 30$ m, ${\Delta }p = 10$ MPa, 6.5 wt.% of $\hbox {H}_2$O).

The duration of the eruption is estimated by considering the variation with time of the eruption rate curve from its initial value until it becomes zero (Fig. 4). In the first set of simulations, the initial overpressure in the chamber is varied in the range between 10–40 MPa while the other parameters are kept fixed ($r = 30$ m, ${\varphi } = 0.1$, 6.5 and 4 wt.% of $\hbox {H}_2$O for silicic and mafic magma respectively). Initial overpressure has a strong effect on the eruption rate, obtaining a maximum value of over 600 $\hbox {m}^3$/s, but has a negligible effect on the eruption duration (cf. Fig. 4a). On the contrary, the conduit radius has a strong effect on both the duration and the eruption rate with the former obtaining a maximum value of 1 year when $r = 20$ m and the latter taking a maximum value of more than 600 $\hbox {m}^3$/s when $r = 40$ m (cf. Fig. 4b). Rather than just using the conservative estimate of $\hbox {H}_2$O mass fraction (6.5 wt.%) for the silicic magma, a range of values between 6.5–12 wt.% was used also for the purpose of investigating the influence of this parameter on the results. Although the mass fraction of volatiles had no effect on the eruption rate, it increased the duration of the eruption from 0.3 to 0.7 years when its value became 12 wt.% (cf. Fig. 4c). The percentage of mafic magma in the chamber is perhaps the least constrained of all the parameters and in the simulations it was varied from 1 to 15%. At this point it should be mentioned that the cooling rate of the mafic magma is inversely proportional to its volume^20^, therefore for ${\varphi } < 0.1$ a faster cooling rate was assumed (10$^{-6}$ K/s) and when ${\varphi } \ge 0.1$ the cooling rate was set to a value one order of magnitude smaller (10$^{-7}$ K/s). Figure 4d shows that the percentage of mafic magma within the given range has negligible effects on both the duration and the eruption rate.

**Figure 5:**
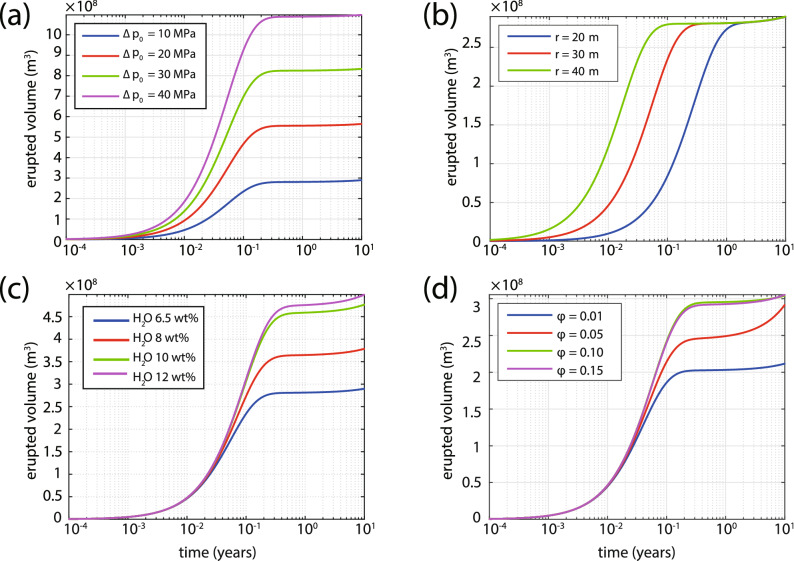
Same as in Fig. 4 but this time depicting erupted volume as a function of time for a range of: (**a**) initial overpressure, (**b**) conduit radius, (**c**) mass fraction of volatiles, and (**d**) percentage of mafic magma.

**Figure 6:**
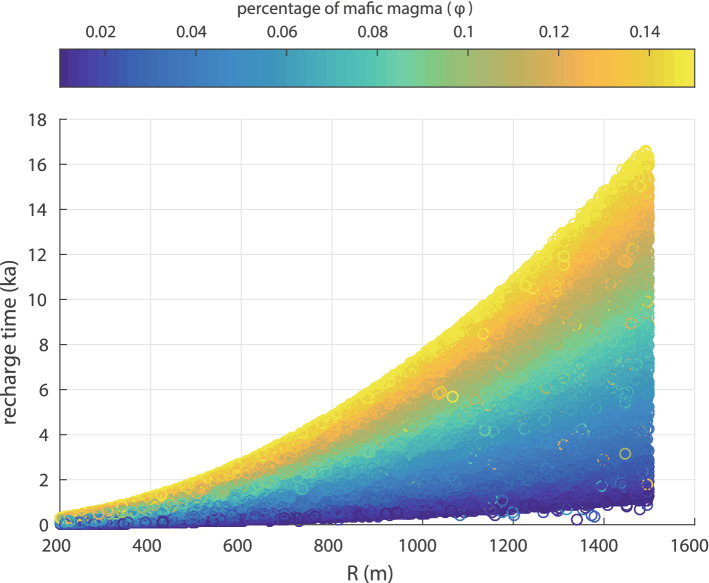
Diagram showing the variation of the recharge time as a function of chamber radius and of the percentage of mafic magma. The coloured circles represent the results of 40,000 Monte Carlo simulations (see text for more details). Note that tomographic images imply a radius of about 1,500 m for the Kolumbo magma chamber.

**Figure 7:**
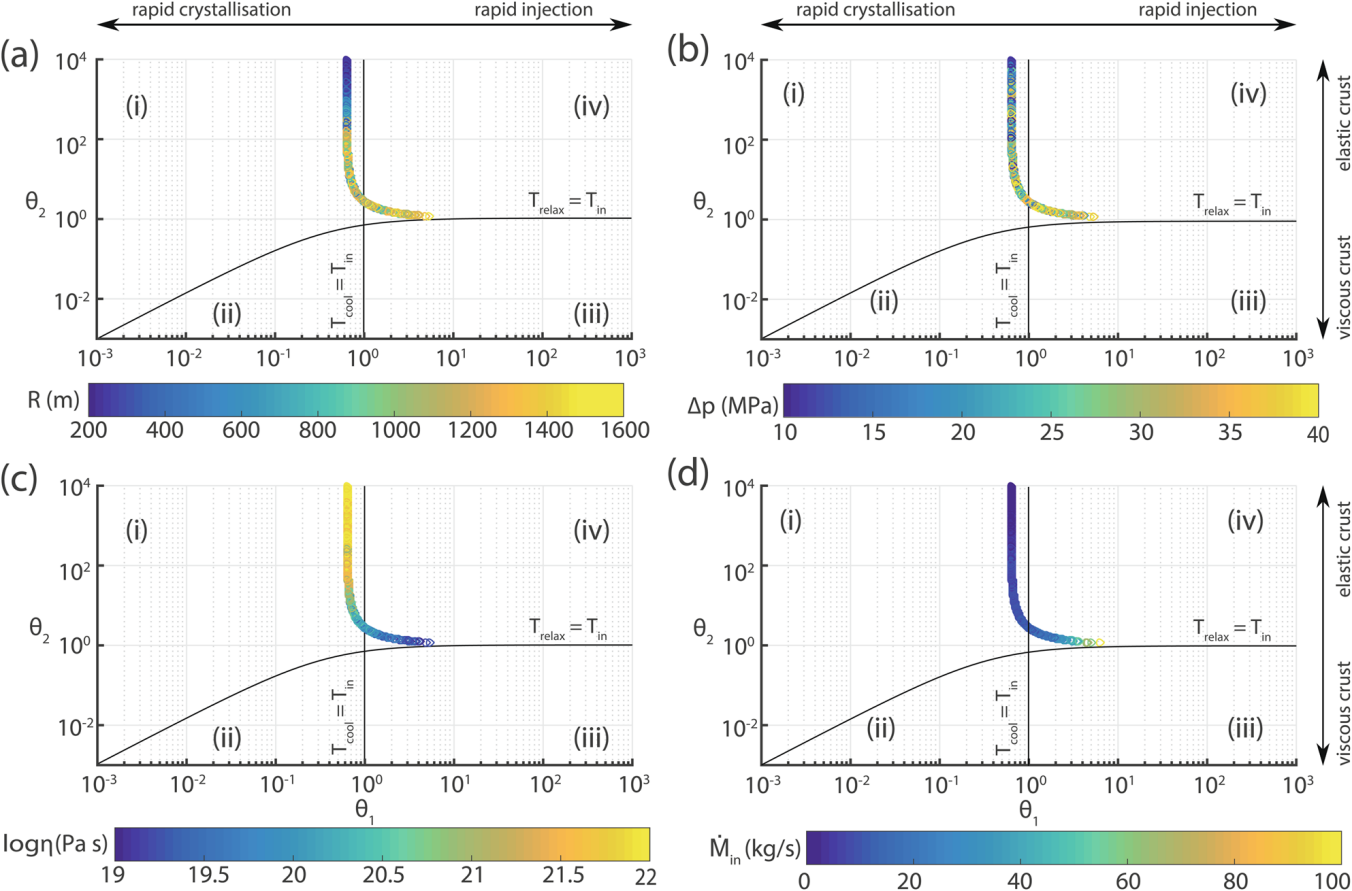
Summary of Monte Carlo simulations for the different magma chamber timescales and ${\theta }_1, {\theta }_2$ proxies as a function of: (**a**) chamber radius *R*, (**b**) overpressure ${\Delta }p$, (**c**) viscosity of the crust ${\eta }$, and (**d**) mass injection rate ${\dot{M}}_{in}$.

The time history of the erupted volume is also calculated for the same range of each parameter and the total volume erupted is estimated by integrating the area under each curve (Fig. 5). The initial value of overpressure seems to have a strong influence on the volume of erupted material with a maximum value of 11 $\hbox {km}^3$ when ${\Delta }p_0$ is equal to 40 MPa (cf. Fig. 5a). When the conduit radius is varied the three curves appear to exhibit a shift in time, but produce essentially the same volume of erupted material (cf. Fig. 5b). The mass fraction of volatiles in the silicic magma also has an important effect on the erupted volume producing a minimum of 2.7 $\hbox {km}^3$ for 6.5 wt.% and a maximum value of 4.6 $\hbox {km}^3$ for 12 wt.% (cf. Fig. 5c). More importantly, simulations show that the percentage of mafic magma that occupies the base of the chamber has a small but visible effect on the erupted volume. Mafic magma corresponding to 1% of the chamber results in an erupted volume of 2 $\hbox {km}^3$ that can reach a value of up to 3 $\hbox {km}^3$ when the percentage of mafic magma becomes 15% (cf. Fig. 5d).

The results of these simulations should be viewed under the prism of observations related to the 1650 AD Kolumbo eruption, and more specifically its duration and erupted volume as outlined previously. The estimated duration of the eruption (0.22–0.76 years) tends to favor a conduit with a radius of about 30 m corresponding to an eruption rate of ${\sim }$ 160 $\hbox {m}^3$/s which can be considered rather low for an eruption that dispersed ash up to western Turkey^19^. However, it has to be noted that the approximation of the conduit shape as a cylinder is a necessary model simplification and that at the initial stages of the eruption the conduit most likely was fissure-like, becoming wider and more cylindrical as the eruption progressed. It is thus quite possible that the eruption started with a smaller size conduit, attaining an eruption rate of several hundred cubic meters per second at later stages when the conduit became larger. A comparison of the total erupted volume during each simulation and the DRE volume of the 1650 AD eruption (${\sim }$ 2 $\hbox {km}^3$) suggests that the initial overpressure should have been around 10 MPa. The simulations also imply that a total erupted volume of 2–2.5 $\hbox {km}^3$ can be produced with the injection of up to 5% of mafic magma in the chamber (cf. Fig. 5d). Based on these results the duration of any future eruption will critically depend on conduit geometry and the mass fraction of volatiles contained in the silicic magma, while the erupted volume will also depend on the percentage of mafic magma injected in the chamber.

### Long-term behaviour: mass injection rate and eruption frequency

A first assessment of the eruption frequency at Kolumbo has been made by Hübscher et al.^15^ who suggested that the deposits from 5 eruptions within a period of > 70 ka imply a recurrence interval of > 10 ka. However, before addressing the issue of eruption recurrence time, an estimate of the mass injection rate of mafic magma ${\dot{M}}_{in}$ has to be made. Mafic magma can be injected in the chamber either episodically, as in the case of the nearby Santorini caldera^21,22^, or through the steady influx of melt. Seismological observations at Kolumbo tend to favour the latter possibility, since precise relative locations of microearthquakes cluster below the magma chamber at depths between 8–15 $\hbox {km}$^23–25^. This seismicity has been recorded at Kolumbo during different time periods indicating that it is not a transient feature, but rather represents the response of brittle rocks to the steady migration of fluids in the magma chamber.

Degruyter and Huber^26^ have formulated a thermomechanical model for the eruption frequency of silicic magma chambers that reside in the upper crust and have also suggested scaling relationships that describe the long-term behaviour of such chambers. One of these relationships connects the number *N* of observed eruptions with the properties of the magma chamber and those of the upper crust such that

1 $$\begin{aligned} N = \frac{(-4{\pi }b_{3}{\sigma }_{mlm}{\Delta }p)R^{3} + (4{\pi }b_{2}{\eta }{\kappa }{\sigma }_{mlm})R + (3b_{1}{\eta }{\dot{M}}_{in})}{(4{\pi }{\eta }{\kappa }{\sigma }_{mlm})R} \end{aligned}$$

where $b_1 = 2.4$, $b_2 = 3.5$, $b_3 = 2.5$ are scaling constants, ${\sigma }_{mlm}$ is the density of mafic melt, ${\eta }$ is the viscosity of the crust, ${\kappa }$ is the thermal diffusivity of the crust and the remaining parameters are the same as previously. This equation can be solved numerically for ${\dot{M}}_{in}$ (taking $N = 5$) and then it is possible to calculate the recharge time needed to fill a magma chamber of radius *R* with a percentage ${\varphi }$ of mafic magma. In order to check how variations in *R*, ${\eta }$, ${\Delta }p$ and ${\varphi }$ affect the calculated values of ${\dot{M}}_{in}$ and recharge time, a total of 40,000 Monte Carlo simulations were performed where each of these parameters was varied randomly and simultaneously within a specific range (for details see section “Methods”). During all calculations parameters ${\sigma }_{mlm}$ and ${\kappa }$ were assumed as constant and their values can be found in Table 1. Based on these simulations the mean value of the mass injection rate is equal to 5.94 kg/s with a standard error of ${\pm }$ 0.01 kg/s, while in ${\sim }$ 93% of the simulations (i.e, 37,587 times) the calculated value of ${\dot{M}}_{in}$ was smaller than 10 kg/s.

The scatter plot in Fig. 6 shows the variation of recharge time as a function of the chamber radius and the percentage of mafic magma. It can be seen that longer recharge times are needed for larger radius chambers and for larger percentages of mafic magma within the chamber. For a chamber size that has a radius between 1,400–1,500 m, the average time needed for injecting 1%, 5%, 10%, 15% of mafic magma is about 1.4, 5.1, 11.3 and 13.7 ka respectively. Considering that less than 400 years have elapsed since the last eruption and even requiring the minimum percentage (1%) of mafic magma to be injected in the chamber, more than 700 years are needed before another eruption can occur. It is interesting to note that the recharge time for 10–15% of mafic magma to be injected in the chamber, is similar to the recurrence interval of more than 10 ka proposed by Hübscher et al.^15^. These results also suggest that an eruption in the next few years is possible only if the chamber radius is smaller than 800 m. Such a chamber size is probably unrealistic for Kolumbo, since it would mean that its volume is equal to or smaller than the DRE volume of the 1650 AD eruption.

### Eruption triggering mechanism

Another question that is related to the long-term behaviour of Kolumbo’s magma chamber has to do with the triggering mechanism of its eruptions. In their model, Degruyter and Huber^26^ defined three timescales that can help classify a magma chamber according to its eruption triggering mechanism and these are

2 $$\begin{aligned} {\tau }_{in}= & {} \frac{4{\pi }}{3} \frac{{\rho }R^3}{{\dot{M}}_{in}} \end{aligned}$$

3 $$\begin{aligned} {\tau }_{cool}= & {} \frac{R^2}{\kappa } \end{aligned}$$

4 $$\begin{aligned} {\tau }_{relax}= & {} \frac{{\eta }}{{\Delta }p} \end{aligned}$$

These timescales are used to form the dimensionless ratios ${\theta }_1 = {\tau }_{cool}/{\tau }_{in}$ and ${\theta }_2 = {\tau }_{relax}/{\tau }_{in}$ that function as proxies for the heat budget of the chamber (i.e, heat loss/supply) and the balance between viscous relaxation of the crust and mass injection rate respectively. Based on these timescales, four eruption triggering regimes can be identified^26^: (i) triggering by second boiling when ${\tau }_{cool} < {\tau }_{relax}$ and ${\theta }_1<$ 1, (ii) no eruptions when ${\tau }_{cool} > {\tau }_{relax}$ and ${\theta }_1<$ 1, (iii) triggering by buoyancy when ${\theta }_2<$ 1 and ${\theta }_1>$ 1, (iv) triggering by mass injection when ${\theta }_2>$ 1 and ${\theta }_1 > 1$.

It is possible to use the randomised quantities (${\dot{M}}_{in}$, *R*, ${\eta }$, ${\Delta }p$) calculated in the previous section in order to repeat the Monte Carlo simulations for the three timescales as well as ${\theta }_1$ and ${\theta }_2$. The simulated values of ${\theta }_1$ and ${\theta }_2$ can then be plotted in a diagram similar to the one used by Degruyter and Huber, where the fields of the four different eruption triggering regimes can be clearly delineated. Figure 7 shows such diagrams in which the simulated values are plotted also as a function of the randomised quantities. The plotted points define trajectories that initially are parallel and very close to the border between regimes (i) and (iv), while under certain conditions ($R > 1{,}200$ m, ${\Delta }p > 25$ MPa and ${\eta } < 10^{20}$ Pa s) they may shift completely inside the field of regime (iv). The chamber radius and the viscosity of the crust around it likely satisfy the two of these conditions, at the same time however, a magma chamber with $R > 1{,}200$ m will probably require smaller rather than larger overpressure as implied also by the simulations of the 1650 AD eruption discussed previously. Hence, Kolumbo’s magma chamber probably lies within the field of regime (i) but quite close to the border with regime (iv). Such a hybrid chamber behaviour can be explained by considering that prior to an eruption the pressure in the chamber increases due to exsolution of volatiles from the mafic magma as it cools and crystallises. This by itself cannot result to an eruption, since the heating of the larger silicic layer will cause resorption of the gas and dissolution of its crystals which will tend to decrease the pressure^27^. A small but steady influx of mafic melt is therefore necessary in order to keep the pressure in the chamber constantly rising.

These processes are also relevant to the way and to the degree that the two types of magma are mixed. The erupted products from the K5 (1650 AD) and K2 layer do not display any evidence of extensive mixing, but the mafic enclaves support magma mingling without any chemical hybridisation^12,17^. This seems to contradict the suggestion of steady influx of mafic melt in the chamber, since in this case greater mixing would be expected that would result in hybrid erupted products. However, large-scale mixing between the two magmas can be suppressed if the mafic magma maintains low viscosity, or its cooling rate is sufficiently small^28^. As the quantity of the mafic magma increases with time its cooling rate should become smaller and bubbles should start rising, forming a bubbly layer at the interface with the silicic magma^28,29^. Mafic enclaves can then be formed when this boundary layer becomes less dense, resulting in a Rayleigh-Taylor instability that would allow the bubbles to create vertical plumes within the layer of silicic magma^9,28,29^.

## Conclusions

Submarine volcanism can be a source of significant hazards for maritime traffic and for nearby islands as it can generate powerful underwater explosions, floating pumice rafts, tsunami and pyroclastic flows. Kolumbo submarine volcano lies at the center of the southern Aegean and is surrounded by inhabited islands whose population increases significantly during the summer months. It is therefore of particular importance for volcanic risk assessment to investigate its potential for future eruptions and make inferences about their duration and erupted volume. This study combined constraints from multi-disciplinary observations (seismological, petrological, geochemical) in order to model the evolution of Kolumbo’s magma chamber during its last eruption in 1650 AD, also with the aim of understanding its long-term eruption behaviour. The main conclusions of this study are as follows:

- Simulations of the evolution of Kolumbo’s magma chamber during the 1650 AD eruption were performed by using a chamber model containing silicic magma that is underlain by a layer of mafic magma. Results showed that the conduit radius and the mass fraction of volatiles have the most significant effect on the eruption duration, while the eruption rate is also influenced by the initial overpressure in the chamber. On the other hand, the total volume erupted depends not only on the initial overpressure and mass fraction of volatiles of the silicic magma, but also on the quantity of mafic magma residing inside the chamber. Using as constraints historical and geological observations it is possible to conclude that the 1650 AD eruption likely involved an initial overpressure of about 10 MPa, a conduit radius of 40–30 m, and mafic magma that occupied up to 5% of the chamber volume.
- For a chamber radius consistent with the tomographic results at Kolumbo the average recharge time varies from 1.4 to 13.7 ka for mafic magma that occupies 1–15% of the chamber volume. This means that an eruption at Kolumbo in the foreseeable future is not likely, unless the magma chamber is much smaller ($R < 800$ m), in which case the recharge time is in the order of a few hundred years. However, such a scenario comes into conflict with the small number of observed eruption cycles at Kolumbo ($N = 5$) and the DRE volume of the deposits of the 1650 AD eruption (${\sim }$2 $\hbox {km}^3$) that would be larger than the volume of the chamber itself.
- Monte Carlo simulations of the ratios that indicate the balance between heat loss versus heat supply and viscous versus elastic behavior of the crust, classify Kolumbo’s magma chamber to a regime where pressure inside the chamber increases by a combination of volatile exsolution from the mafic magma and by a small but steady influx of mafic melt.

Although the results of this study suggest that the probability of an eruption at Kolumbo in the near future is rather low, there are still potential hazards related to Kolumbo’s intense hydrothermal activity. The most important of these hazards is the constant degassing of $\hbox {CO}_2$ and its accumulation near the bathymetric low defining Kolumbo’s crater^30,31^. The progressive buildup of $\hbox {CO}_2$ concentration in the seawater increases the chances of an episodic overturn that would result in the release of large quantities of gas in the surface, potentially suffocating the nearby islands. It is therefore prudent to closely monitor Kolumbo’s activity and study further its hydrothermal system in order to fully understand and mitigate the risk posed by this submarine volcano.

## Methods

### Model equations

The first equation describes the rate of change of pressure in the magma chamber as a response to the eruption output, as well as the heat transfer between the two magma types^27^

5 $$\begin{aligned} \frac{d{\Delta }p}{dt} = \frac{1}{f} \left( -\frac{Q}{{\rho }_s} + W_{m} \frac{dT_m}{dt} + W_{s} \frac{dT_s}{dt}\right) \end{aligned}$$

where ${\Delta }p$ is the chamber overpressure defined as the difference between chamber pressure and lithostatic pressure, *Q* is the mass eruption rate, ${\rho }_s$ is the density of the silicic magma, $W_m$ and $W_s$ are quantities that describe the rate of change of volume with temperature of mafic magma ($T_m$) and temperature of silicic magma ($T_s$) respectively. The quantity *f* represents the effective chamber volume divided by the effective compressibility for each magma type (${\beta }_s$, ${\beta }_m$) such that $f = f_{m} + f_{s} + f_{w}$, while $f_w$ is equal to $V/{\beta }_{w}$ where *V* is the chamber volume and ${\beta }_w$ is the bulk modulus of the rock surrounding the chamber (see Woods and Huppert^27^ for equations describing $W_m, W_s, f_m, f_s$).

The magma chamber is connected to the surface with a conduit of radius *r* which here is represented as cylindrical rather than a rectangular fracture (cf. Fig. 3). This geometry avoids including in the model extra parameters, such as fracture width and aperture, that are poorly known. Since the focus of this work is not the phenomena that took place near the erupting vent, a simple expression for the eruption mass flow rate *Q* is utilized that is given by^32^

6 $$\begin{aligned} Q = \frac{{\rho }_{s}SA^{2}{\Delta }p}{{\mu }_{s}H} \end{aligned}$$

where S is the shape factor of the conduit (${\sim }$ 0.1), *A* ($={\pi }r^{2}$) is the cross-sectional area of the conduit, and ${\mu }_{s}$ is the viscosity of the silicic magma that is considered a constant. The second equation that completes the model description represents the mass decrease rate of the silicic magma during the eruption as

7 $$\begin{aligned} \frac{dM_s}{dt} = -Q \end{aligned}$$

Equations (5)–(7) form a system of ordinary differential equations with respect to time that can be solved to understand the behaviour of Kolumbo’s magma chamber during the 1650 AD eruption, as well as to draw conclusions about the duration and volume of future eruptions. The system of ordinary differential equations was numerically integrated at a time step of 1 minute using Matlab’s *ode15s* function which is a variable order solver suitable for stiff systems^33^.

### Magma density calculation

The bulk density of both the mafic and the silicic magma can be calculated by considering them as mixtures of melt, crystals and volatiles in which case the density is^34^

8 $$\begin{aligned} {\rho }_{m,s} = \left( \frac{n_{m,s}}{{\rho }_g} + \frac{1 - n_{m,s}}{{\sigma }_{m,s}}\right) ^{-1} \end{aligned}$$

where $n_{m,s}$ is the fraction of exsolved volatiles in each magma type, ${\rho }_g$ is the density of $\hbox {H}_2$O vapor obeying the perfect gas law, and ${\sigma }_{m,s}$ is the bulk density of melt and its crystals. The latter quantity can be further calculated by the following expression^34^

9 $$\begin{aligned} {\sigma }_{m,s} = \left( \frac{x_{m,s}}{{\sigma }_{c(m,s)}} + \frac{1 - x_{m,s}}{{\sigma }_{ml(m,s)}}\right) ^{-1} \end{aligned}$$

where $x_{m,s}$ is the mass fraction of crystals in the magma, ${\sigma }_c$ is the density of the crystals, and ${\sigma }_{ml}$ is the density of the melt. In the model simulations the mafic magma contains no crystals, hence $x_m =$ 0.

### Heat transfer equations

The temperature of mafic magma is decreasing as heat is transferred to the silicic magma and the rate of change occurs according to the relation^27^

10 $$\begin{aligned} \frac{dT_m}{dt} = -G \end{aligned}$$

where *G* represents the cooling rate of the mafic magma. On the contrary, the temperature of the silicic magma is increasing as it receives more heat and the rate of change of its temperature is given by

11 $$\begin{aligned} M_s (Cp_{s} + L{\Gamma }_{s}) \frac{dT_s}{dt} = (Cp_{m} + L{\Gamma }_{m})GM_{m} \end{aligned}$$

where $M_s$ and $M_m$ are the masses of silicic and mafic magma, $Cp_{s}$ and $Cp_{m}$ are the specific heat for each magma type, *L* is latent heat of crystallisation, ${\Gamma }_s$ and ${\Gamma }_m$ are the crystallisation rates per degree of cooling for the two magma types.

### Monte Carlo simulations

Randomised values of a quantity *X* can be calculated by the repeated use of the following equation

12 $$\begin{aligned} X_r = X_{min} + (X_{max}-X_{min}){\times }rand \end{aligned}$$

where $X_r$ is the randomised value, $X_{max}$ and $X_{min}$ are the maximum and minimum values of *X* considered, while *rand* is a random number created by using Matlab’s random number generator. For the radius *R* of the magma chamber the chosen range was 200–1500 m, for the overpressure ${\Delta }p$ it was 10–40 MPa, and for the viscosity of the crust ${\eta }$ this was 10$^{19}$–10$^{22}$ Pa s. The percentage of mafic melt ${\varphi }$ followed the range of 0.01–0.15 (1–15%).

## Data Availability

No datasets were analysed during the current study.

## References

[1] Cashman KV, Sparks RS (2013). How volcanoes work: a 25 years perspective. Bull. Geol. Soc. Am..

[2] Bachmann O, Huber C (2016). Silicic magma reservoirs in the Earth’s crust. Am. Mineral..

[3] Edmonds M, Cashman KV, Holness M, Jackson M (2019). Architecture and dynamics of magma reservoirs. Philos. Trans. R. Soc. A.

[4] Sparks SRJ, Annen C, Blundy JD, Cashman KV, Rust AC, Jackson MD (2019). Formation and dynamics of magma reservoirs. Philos. Trans. R. Soc. A.

[5] Huber C, Townsend M, Degruyter W, Bachmann O (2019). Optimal depth of subvolcanic magma chamber growth controlled by volatiles and crust rheology. Nat. Geosci..

[6] Sparks SRJ, Sigurdson H, Wilson L (1977). Magma mixing: a mechanism for triggering acid explosive eruptions. Nature.

[7] Huppert HE, Sparks RSJ, Turner JS (1982). Effects of volatiles on mixing in calc-alkaline magma systems. Nature.

[8] Folch A, Martí J (1998). The generation of overpressure in felsic magma chambers by replenishment. Earth Planet. Sci. Lett..

[9] Plail M, Edmonds M, Woods AW, Barclay J, Humphreys MCS, Heard RA, Christopher T (2018). Mafic enclaves record syn-eruptive basalt intrusion and mixing. Earth Planet. Sci. Lett..

[10] Floyd MA, Billiris H, Paradisis D, Veis G, Avallone A, Briole P, McClusky S, Nocquet J-M, Palamartchouk K, Parsons B, England PC (2010). A new velocity field for Greece: implications for the kinematics and dynamics of the Aegean. J. Geophys. Res..

[11] Papazachos BC, Karakostas VG, Papazachos CB, Scordilis EM (2000). The geometry of the Wadati–Benioff zone and lithospheric kinematics in the Hellenic arc. Tectonophysics.

[12] Klaver M, Carey S, Nomikou P, Smet I, Godelitsas A, Vroon P (2016). A distinct source and differentiation history for Kolumbo submarine volcano, Santorini volcanic field, Aegean arc. Geochem. Geophys. Geosyst..

[13] Dimitriadis I, Papazachos C, Panagiotopoulos D, Hatzidimitriou P, Bohnhoff M, Rische M, Meier T (2010). P and S velocity structures of the Santorini–Columbo volcanic system (Aegean Sea, Greece) obtained by non-linear inversion of travel times and its tectonic implications. J. Volcanol. Geotherm. Res..

[14] Konstantinou KI, Yeh T-Y (2012). Stress field around the Coloumbo magma chamber, southern Aegean: its significance for assessing volcanic and seismic hazard in Santorini. J. Geodyn..

[15] Hübscher C, Ruhnau M, Nomikou P (2015). Volcano-tectonic evolution of the polygenetic Kolumbo submarine volcano/Santorini (Aegean Sea). J. Volcanol. Geotherm. Res..

[16] Dominey-Howes DTM, Papadopoulos GA, Dawson AG (2000). Geological and historical investigation of the 1650 Mt Columbo (thera island) eruption and tsunami, Aegean Sea, Greece. Nat. Hazards.

[17] Cantner K, Carey S, Nomikou P (2014). Integrated volcanologic and petrologic analysis of the 1650 AD eruption of Kolumbo submarine volcano, Greece. J. Volcanol. Geotherm. Res..

[18] Ulrova M, Paris R, Nomikou P, Kelfoun K, Leibrandt S, Tappin DR, McCoy FW (2016). Source of the tsunami generated by the 1650 AD eruption of Kolumbo submarine volcano (Aegean Sea, Greece). J. Volcanol. Geotherm. Res..

[19] Fuller S, Carey S, Nomikou P (2018). Distribution of fine-grained tephra from the 1650 CE subinarine eruption of Kolumbo volcano Greece. J. Volcanol. Geotherm. Res..

[20] Snyder D (2000). Thermal effects of the intrusion of basaltic magma into a more silicic magma chamber and implications for eruption triggering. Earth Planet. Sci. Lett..

[21] Martin VM, Morgan DJ, Jerram DA, Caddick MJ, Prior DJ, Davidson JP (2008). Bang! Month-scale eruption triggering at Santorini Volcano. Science.

[22] Druitt TH, Costa F, Daloule E, Dungan M, Scaillet E (2012). Decadal to monthly timescales of magma transfer and reservoir growth at a caldera volcano. Nature.

[23] Bohnhoff M, Rische M, Meier T, Becker D, Stavrakakis G, Harjes HP (2006). Microseismic activity in the Hellenic Volcanic Arc, Greece, with emphasis on the seismotectonic setting of the Santorini Amorgos Zone. Tectonophysics.

[24] Dimitriadis I, Karagianni E, Panagiotopoulos D, Papazachos C, Hatzidimitriou P, Bohnhoff M, Rische M, Meier T (2009). Seismicity and active tectonics at Coloumbo Reef (Aegean Sea, Greece): Monitoring an active volcano at Santorini Volcanic Center using a temporary seismic network. Tectonophysics.

[25] Konstantinou KI, Evangelidis CP, Liang W-T, Melis NS, Kalogeras I (2013). Seismicity, Vp/Vs and shear wave anisotropy variations during the 2011 unrest at Santorini caldera, southern Aegean. J. Volcanol. Geotherm. Res..

[26] Degruyter W, Huber C (2014). A model for eruption frequency of the upper crustal silicic magma chambers. Earth Planet. Sci. Lett..

[27] Woods AW, Huppert HE (2003). On the magma chamber evolution during slow effusive eruptions. J. Geophys. Res..

[28] Philips JC, Woods AW (2002). Suppression of large-scale magma mixing by melt-volatile separation. Earth Planet. Sci. Lett..

[29] Thomas N, Tait S, Koyaguchi T (1993). Mixing of stratified liquids by the motion of gas bubbles: application to magma mixing. Earth Planet. Sci. Lett..

[30] Carey S, Nomikou P, CroffBell K, Lilley M, Lumpton J, Roman C, Stathopoulou E, Bejelou K, Ballard R (2013). $$\text{CO}_2$$ degassing from hydrothermal vents at Kolumbo submarine vocano, Greece, and the accumulation of acidic crater water. Geology.

[31] Rizzo AL, Caracausi A, Chavagnac V, Nomikou P, Polymenakou P, Mandalakis M, Kotoulas G, Magoulas A, Castillo A, Lampridou D, Marusczak M, Sonke JE (2019). Geochemistry of $$\text{ CO}_2$$-rich gases venting from submarine volcanism: The case of Kolumbo (Hellenic Volcanic Arc, Greece). Front. Earth Sci..

[32] Stasiuk M, Sparks RSJ, Jaupart C (1993). On the variations of flow rate in non-explosive lava eruptions. Earth Planet. Sci. Lett..

[33] Shampine LF, Reichelt MW (1997). The Matlab ODE suite. SIAM J. Sci. Comput..

[34] Huppert HE, Woods AW (2002). The role of volatiles in magma chamber dynamics. Nature.

